# Peripheral capillary non-perfusion in asymptomatic Waldenström's macroglobulinemia

**DOI:** 10.1186/1471-2415-10-30

**Published:** 2010-12-03

**Authors:** Chryssanthi Koutsandrea, Athanasios Kotsolis, Ilias Georgalas, Dimitris Papaconstantinou, Ioannis Ladas

**Affiliations:** 1 Department of Ophthalmology " G.Gennimatas" Hospital, University of Athens, Athens, Greece

## Abstract

**Background:**

To report the rare association of peripheral retinal ischemia in a patient with Waldenström's macroglobulinemia.

**Case Presentation:**

A 39-year old man with a recent diagnosis of asymptomatic Waldenström's macroglobulinemia (WM) was referred from his physician for ocular evaluation. The fundus examination in his right eye (RE) revealed very mild central vein dilation, while retinal hemorrhages associated with microaneurismal alterations of the vascular plexus were detected at the temporal periphery. Fluoroscein angiography of his RE revealed an extended area of capillary dropout distal to the microaneurismal lesions. In our patient with WM an extensive area of capillary non-perfusion, in the absence of severe involvement of the posterior pole was documented; this association to the best of our knowledge has never been reported before.

**Conclusion:**

Although the incidence of the disease is rare, meticulous examination of the retinal periphery should be performed in all patients with WM and vice versa the differential diagnosis of peripheral retinal ischemia of unknown origin should include an investigation to rule out asymptomatic Waldenström's macroglobulinemia.

## Backround

Waldenström's macroglobulinemia (WM) is a non-Hodgkin's B-cell lymphoplasmocytic lymphoma characterized by bone marrow infiltration and production of monoclonal IgM[1]. Patients with WM may be asymptomatic and diagnosed by chance (asymptomatic WM) or suffer from fever, weight loss, lymphadenopathy, organomegaly, gastrointestinal bleeding, heart failure, and neuropathy[1].

The increased concentration of proteins may lead to the development of hyperviscosity syndrome (HVS) which is the main reason of central retinal disorders like vascular occlusion and hemorrhages in patients with WM[2-4]. We report a case where the development of peripheral retinal hemorrhages was associated with an extended area of capillary non-perfusion in the retinal periphery of a patient with asymptomatic WM which to the best of our knowledge has never been reported before.

## Case presentation

**Figure 1:**
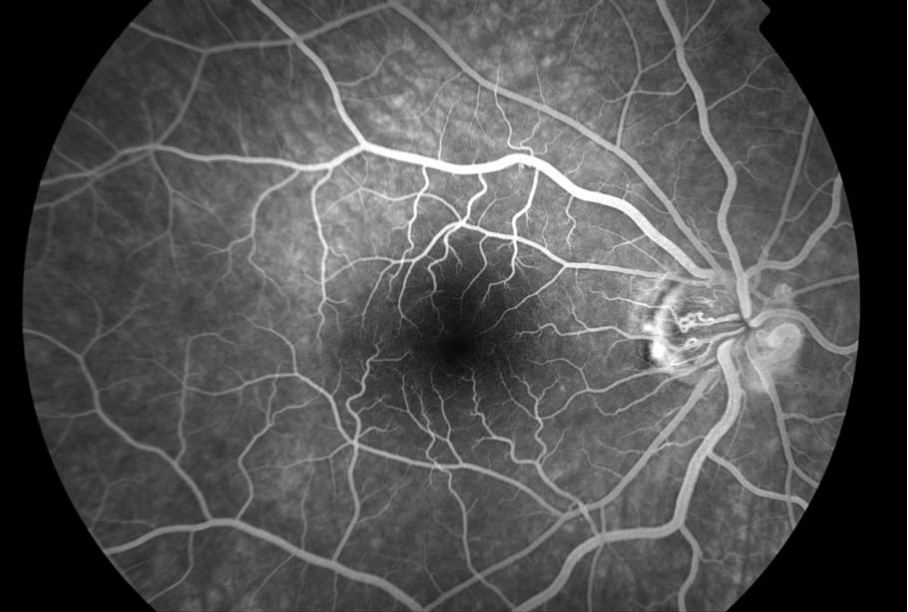
**Fluorescein angiography of the posterior pole of the right eye showing only mild central vein dilation**.

**Figure 2:**
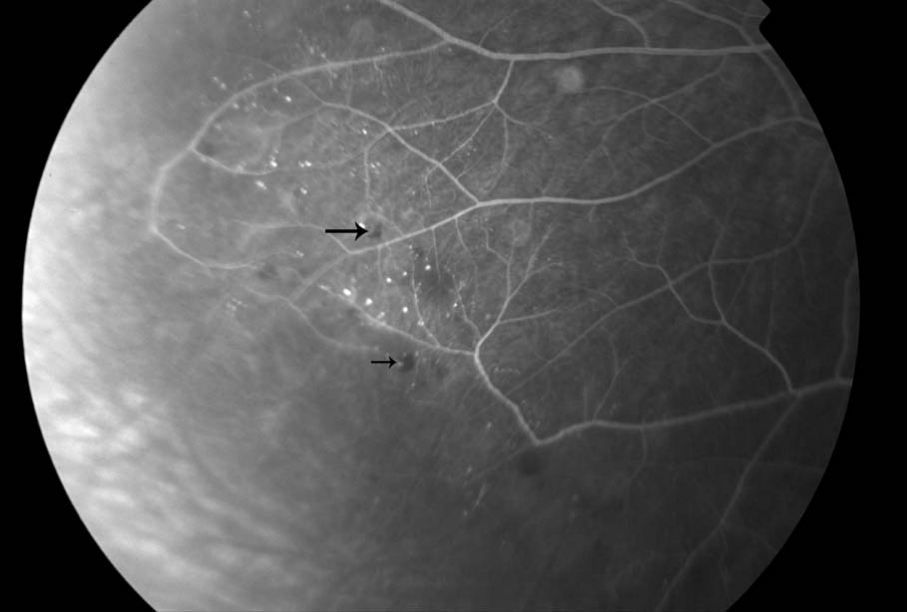
**Fluorescein angiography of the right eye demonstrating an extended area of capillary non-perfusion distal to the microaneurismal lesions**.

A 39-year old male with a recent diagnosis of asymptomatic Waldenström's macroglobulinemia confirmed with bone marrow biopsy (IgM: 2.3 g/L, total protein 10.4 gr/dl) was referred from his internist, to our clinic for ocular evaluation. The patient had a history of vitrectomy for retinal detachment in his left eye in 2003. Both eyes of the patient, were highly myopic (13D OU). His visual acuity was 20/20 RE and 20/30 LE. Anterior segment examination was unremarkable. The intraocular pressure was 14 mmHg in the RE and 12 mmHg in the LE. Fundus examination of his RE revealed very mild central vein dilation associated with peripheral (mainly temporally) retinal hemorrhages and microaneurismal alterations of the vascular bed. Examination of his LE revealed peripapillary atrophy, mild retinal pigment epithelium (RPE) alterations of the fovea, and an area of peripheral chorioretinal atrophy due to cryopexia. Fluorescein angiography of his RE demonstrated an extended area of capillary non-perfusion distal to the microaneurismal lesions (Figure 1, 2).

## Conclusions

The most frequent reported retinal findings in hyperviscosity syndrome are central retinal hemorrhages and vein dilation[2,3]. Menke et al^2 ^have recently reported that these changes represent the more/most severe findings, but not necessarily the main findings. Venous dilation seem to be the first signs of HVS followed by peripheral retinal hemorrhages, which can only be observed by indirect ophthalmoscopy. Only in advanced HVS one can find central venous dilation, increased tortuosity, central hemorrhages, disc edema, and venous sausaging[2]. The authors emphasized the importance of indirect ophthalmoscopy with scleral depression for the evaluation of these earliest structural damages but did not provide any information regarding the peripheral vascular bed.

Fluoroscein angiography, in our case, revealed an extensive area of peripheral capillary dropout associated with microaneurysms and retinal hemorrhages (Figure 1, 2). Non-perfusion of the peripheral capillary bed can be explained on the basis of hyperviscosity in WM. Monoclonal IgM, which are secreted in high concentration, form aggregates and bind water through their carbohydrate component resulting in an increase in the resistance to blood flow and impaired transit through the microcirculation.^1^

In our case, peripheral capillary ischemia and hemorrhages were associated with only a very mild central vein dilation showing that microcirculation may, at least in some cases, be more susceptible and involved earlier than macrocirculation in patients with HVS. Especially in patients with low levels of IgM, evaluation of the peripheral vascular plexus may reveal early signs of HVS in otherwise asymptomatic patients. The fact that our patient was highly myopic should be also taken into consideration; it is not unlikely, that capillary meshwork is more susceptible to damage or to occlusion in patients with high myopia and hyperviscosity syndromes. Additionally, capillary non perfusion would be expected to be bilateral, however, this could not be evaluated in the fellow eye of our patient, where extensive cryotherapy had been applied during vitrectomy for the treatment of the retinal detachment.

In our patient, with asymptomatic WM, the peripheral retinal hemorrhages were associated with an extensive area of capillary non-perfusion, in the absence of severe involvement of the posterior pole; this association to the best of our knowledge has never been reported before. Although the incidence is rare, meticulous examination of the retinal periphery should be performed in all patients with WM and vice versa the differential diagnosis of peripheral retinal ischemia of unknown origin should include an investigation to rule out asymptomatic Waldenström's macroglobulinemia.

## Consent

Written informed consent was obtained from the patient for publication of this case report and any accompanying images. A copy of the written consent is available for review by the Editor-in-Chief of this journal.

## Competing interests

The authors declare that they have no competing interests.

## Authors' contributions

CK, IG and AK were in charge of the medical care of the patient and performed the different surgeries. DP, IL were responsible for the photographs and the literature review. CK, IG and AK wrote the manuscript. All authors reviewed it, drafted it critically and provided helpful comments. All authors read and approved the final manuscript.

## Pre-publication history

The pre-publication history for this paper can be accessed here:

http://www.biomedcentral.com/1471-2415/10/30/prepub
